# A new microfluidic method enabling the generation of multi-layered tissues-on-chips using skin cells as a proof of concept

**DOI:** 10.1038/s41598-021-91875-z

**Published:** 2021-06-23

**Authors:** L. Valencia, V. Canalejas-Tejero, M. Clemente, I. Fernaud, M. Holgado, J. L. Jorcano, D. Velasco

**Affiliations:** 1 Department of Bioengineering and Aerospace Engineering, Universidad Carlos III de Madrid (UC3M), Madrid, Spain; 2 Division of Epithelial Biomedicine, CIEMAT, Madrid, Spain; 3 Group of Optics, Photonics and Biophotonics (GOFB), Center for Biomedical Technology, Universidad Politécnica de Madrid, Madrid, Spain; 4 Departamento de Física Aplicada e Ingeniería de Materiales, Escuela Técnica Superior de Ingenieros Industriales, Madrid, Spain; 5 Laboratorio Cajal de Circuitos Corticales, Center for Biomedical Technology, Universidad Politécnica de Madrid and and Consejo Superior de Investigaciones Científicas, C.S.I.C, Campus de Montegancedo, Madrid, Spain; 6 Group of Organ and Tissue on-a-chip and In-Vitro Detection, Health Research Institute of the Hospital Clínico San Carlos, Madrid, Spain; 7 Instituto de Investigación Sanitaria Gregorio Marañón, Madrid, Spain

**Keywords:** Biomedical engineering, Fluidics, Lab-on-a-chip

## Abstract

Microfluidic-based tissues-on-chips (TOCs) have thus far been restricted to modelling simple epithelia as a single cell layer, but likely due to technical difficulties, no TOCs have been reported to include both an epithelial and a stromal component despite the biological importance of the stroma for the structure and function of human tissues. We present, for the first time, a novel approach to generate 3D multilayer tissue models in microfluidic platforms. As a proof of concept, we modelled skin, including a dermal and an epidermal compartment. To accomplish this, we developed a parallel flow method enabling the deposition of bilayer tissue in the upper chamber, which was subsequently maintained under dynamic nutrient flow conditions through the lower chamber, mimicking the function of a blood vessel. We also designed and built an inexpensive, easy-to-implement, versatile, and robust vinyl-based device that overcomes some of the drawbacks present in PDMS-based chips. Preliminary tests indicate that this biochip will allow the development and maintenance of multilayer tissues, which opens the possibility of better modelling of the complex cell–cell and cell–matrix interactions that exist in and between the epithelium and mesenchyme, allowing for better-grounded tissue modelling and drug screening.

## Introduction

In recent years, there has been great scientific and industrial interest in the development of in vitro-engineered three-dimensional (3D) tissue substitutes that more closely mimic human tissues for the testing of cosmetic and pharmaceutical products^1–3^. In this context, bilayered substitutes containing a stromal and an epithelial omponent have been developed to model stratified epithelia such as skin, cornea and oral mucosa^4–7^. These 3D in vitro models also enable emulation of the tumour microenvironment, thus allowing better-founded tumour growth and invasion studies to be performed^8–11^.

The production of this type of 3D bilayered substitute, either manually or by 3D bioprinting, usually involves the use of a collagen or fibrin 3D hydrogel matrix filled with autologous or allogeneic fibroblasts (stromal layer), and autologous or allogeneic keratinocytes seeded on top of it (epithelial layer)^9,12–14^. However, these models are typically cultured under static conditions, which makes them unable to faithfully represent human physiological conditions. Recent interest has focused on generating in vitro 3D tissue models, such as skin and gut, in cell culture insert (CCI) formats with dynamic perfusion. These platforms enable a more physiological transport of nutrients, permitting a more reliable evaluation of drug candidates in terms of toxicity, efficacy and delivery^15–19^. They have also been shown to be able to maintain the viability of these tissue equivalents for longer times than traditional 3D cultures. Given the importance of having bioengineered skin for its clinical applications, particularly in the field of wound healing, in the testing of cosmetics and drugs, and in contributing to the long-lasting interest in achieving an effective transdermal application of drugs, these dynamic CCI cultures have been used to model complex 3D dermo-epidermal equivalents. For example, they have incorporated vascularization, cyclic stress, immune cells or more than a tissue. They have also been used in preliminary drug testing assays (as recent reviews, see^20,21^). Although they are often referred to as tissues-on-a-chip, these systems cannot be considered *stricto *sensu as microfluidic tissues-on-chips, as these are currently defined in the field^22–26^. For instance, in general, tissues are not generated in hollow microchannels, and frequently, they are seeded from the top instead of being microfluidically loaded^27^. Microfluidic tissues-on-chips (TOCs) have thus far modelled simple epithelia, such as lung, gut and liver, or stratified epithelia, such as cornea, as 2D single-cell layers^28–38^, or in the case of skin, as 2D epithelial and dermal cell layers separated by a porous membrane^30,39,40^. Although TOCs represent an important leap towards the generation of improved in vitro epithelial models, challenges such as downscaling preserving tissue function or the lack of a biologically meaningful stromal component still await solution^23^. In this regard, since many tumours are of epithelial origin (i.e., carcinomas), it is of great importance to work up epithelia with a stromal component to take into consideration the well-known role of the microenvironment in tumorigenesis. Another caveat is that, in all these studies, the epithelial basement membrane is usually mimicked by a porous membrane that possesses neither the physicochemical nor the mechanical properties of the native tissue matrix.

From a technical point of view, the vast majority of these microfluidic devices have been developed using photolithography and PDMS^22,41^. However, PDMS-based TOCs still present several challenges and drawbacks. On the one hand, problems at the cell culture and product testing levels are mostly due to the highly hydrophobic character of PDMS^42,43^. On the other hand, the fabrication complexity makes access to this technology difficult to laboratories that lack microfabrication capacities, as is the case for most biological laboratories. Therefore, there is a current demand to design new types of TOCs that overcome the mentioned issues^44,45^. For a recent review, see^46^.

To our knowledge, there is currently no literature describing a microfluidic chip system including both an epithelial and a stromal component. One reason for this is that the rheological behaviour of the hydrogels and the dimensions of the chips make it difficult to generate of a stromal compartment with a determined and homogeneous height along the channel by means of a microfluidic flow. To solve this problem, we designed a way to generate bilayer tissue through parallel flow controlled with syringe pumps. We also present a new cost-effective, robust, vinyl-based microfluidic platform for tissue-on-a-chip applications. This platform is produced by micromachining, which provides more simplicity in the fabrication process, as well as increased flexibility and versatility in the layout of the device (see, for example, references^44,45^). As a proof of concept, we applied our method to skin as a model tissue due to the vast experience of our laboratory in skin tissue engineering and 3D bioprinting ^47–51^. Although it is described here to accommodate bilayer tissue, it can be easily scaled up to model multilayer tissues.

**Figure 1:**
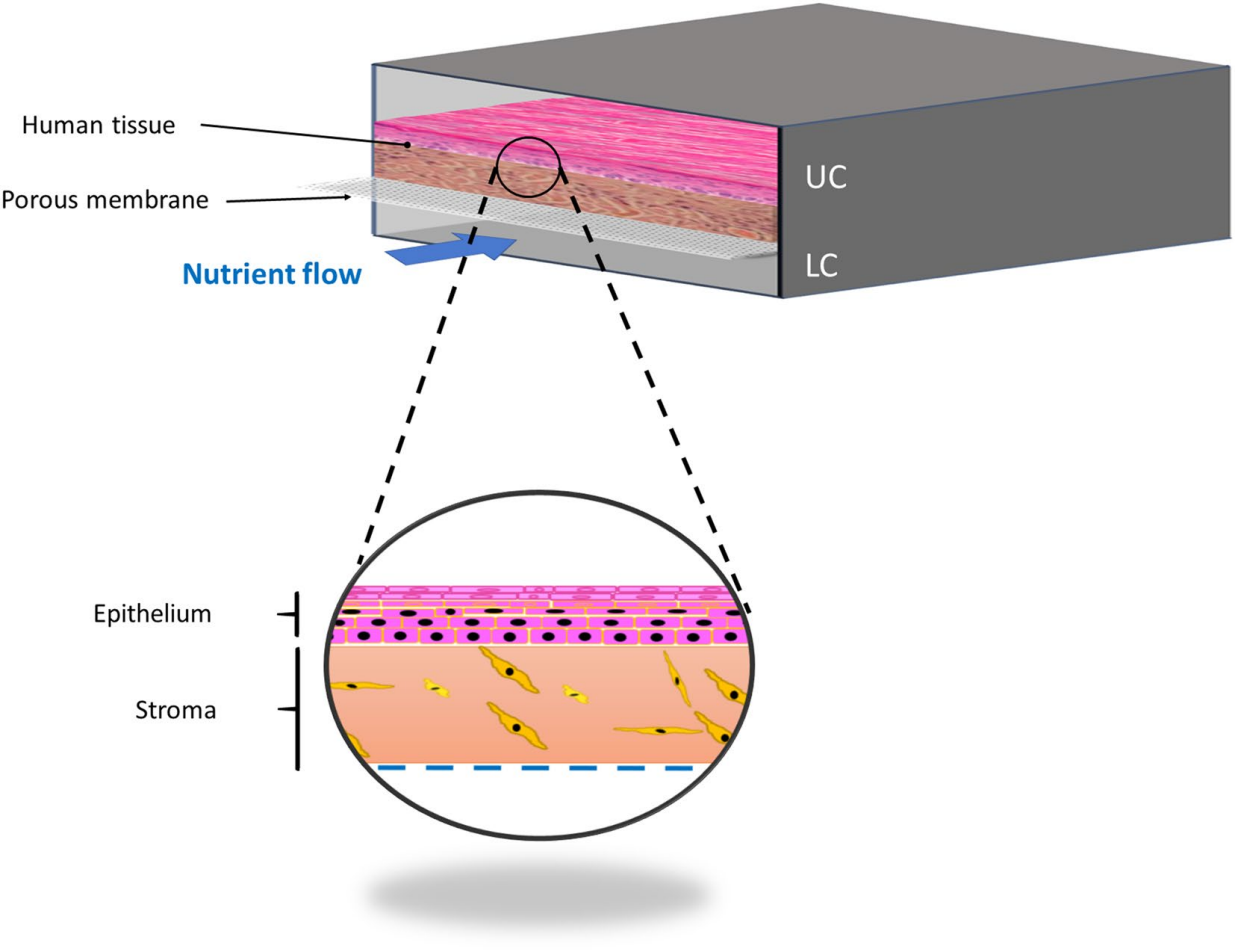
Cross section of the bilayer tissue-on-a-chip chambers and a close view of a human tissue composed of an epithelial and a stromal component, both found in the UC, that is separated from the LC by a porous membrane. There is enough space in the UC for a flow of culture medium, air, or drugs. In the lower chamber, there is a flow to nurture the culture (nutrient flow).

The core of the designed chip (Fig. 1) is composed of two parallel fluidic chambers separated by a microporous polycarbonate (PC) membrane (Merck™) with a 5 µm pore size that allows the interchange of solutes between the lower chamber (LC) and the upper chamber (UC). The UC holds the bilayer tissue, which, in our case, is a human epithelium with an epithelial and a stromal component. Its height is established according to the characteristics of the epithelium (simple, stratified, cornified, etc.). We used the lowest dermal height found in the human body (500 µm) to support epidermal growth and differentiation. The UC size must ensure the correct formation of the epithelium and the circulation of air or drugs. The LC was continuously perfused with culture medium to support the correct function of the tissue. Additionally, other cell types can be grown as a confluent monolayer below the porous membrane to mimic the function of a blood vessel.

## Materials and methods

### Cell culture and fibrin pregel preparation with human dermal fibroblasts

Immortalized human skin keratinocytes (hKCs, HaCaT cell line) and primary human dermal fibroblasts (hFBs) were used to generate a dermo-epidermal model and test the device functionality. hKCs were modified^52^ to express a hybrid histone H2B-GFP protein, providing their nuclei with green fluorescence (H2B-GFP was a gift from Geoff Wahl (Addgene plasmid #11680; http://n2t.net/addgene:11680). hFbs-GFP and HaCaT-RFP were transformed with the vectors pLZRS-IRES-EGFP and pLZRS-IRES-ERFP, respectively, to express cytoplasmic green and red fluorescence^53^. Both fluorescent cell types were used to visualize the stromal and epithelial layers in the UC by fluorescence microscopy using a Zeiss LSM 710 equipped with an Axiovert confocal microscope or a Leica Dmi8 inverted microscope. Primary hFBs obtained from the collections of biological samples of human origin registered in the ‘Registro Nacional de Biobancos para Investigación Biomédica del Instituto de Salud Carlos III’. Both normal and fluorescent cells were kindly donated by Dr. Marta García (UC3M) and cultured in DMEM (Invitrogen Life Technologies) supplemented with 10% FBS (foetal bovine serum, Thermo Scientific HyClone) and 1% antibiotic/antimycotic (Thermo Scientific HyClone). On the day of the experiment, cells were washed with phosphate-buffered saline (PBS) and then incubated with 1 mL of trypsin/EDTA (Sigma Aldrich) for 10 min at 37 °C. Then, 1 mL of supplemented culture medium was added to inactivate the trypsin, centrifuged and resuspended at the desired concentration^47^.

Human fibrinogen (Sigma Aldrich) was used to form a fibrin hydrogel to generate the stromal compartment at a final concentration of 3.5 mg/mL, similar to that found in human blood. Thrombin vials (Sigma Aldrich) contained 10 National Institute of Health (NIH) units. Amchafibrin (Rottafarm) was used as an antifibrinolytic agent. To activate thrombin, 1 mL of CaCl_2_ (1% w/v in saline) was added to each thrombin vial. To prepare a volume of 500 µL of pregel (fibrinogen solution that has not gelled yet), 29.5 µL of activated thrombin, 29.5 µL of Amchafibrin and 382 µL of culture medium containing 200,000 hFBs and 59 µL of fibrinogen (20 mg/mL in saline (0,9% w/v NaCl) were mixed (protocol modified from^47,49^).

### Study of the injectability

Prior to any experiment within the chip, the injectability of the fibrin pregel solution was studied. Rheology tests were performed in a controlled stress rheometer (TA Instruments/AR G2) using a standard aluminium parallel plate (40 mm H/A-Al-998356). Tests were performed at room temperature and using a calibration time of 15 s, and the effect of the shear rate on pregel viscosity was characterized over a range of 1000 to 0.1 s^−1^ with hysteresis. Each pre-gel sample (n = 4) was gently loaded onto the Peltier plate using a pipette.

### Fabrication of a vinyl-based microfluidic chip

**Figure 2:**
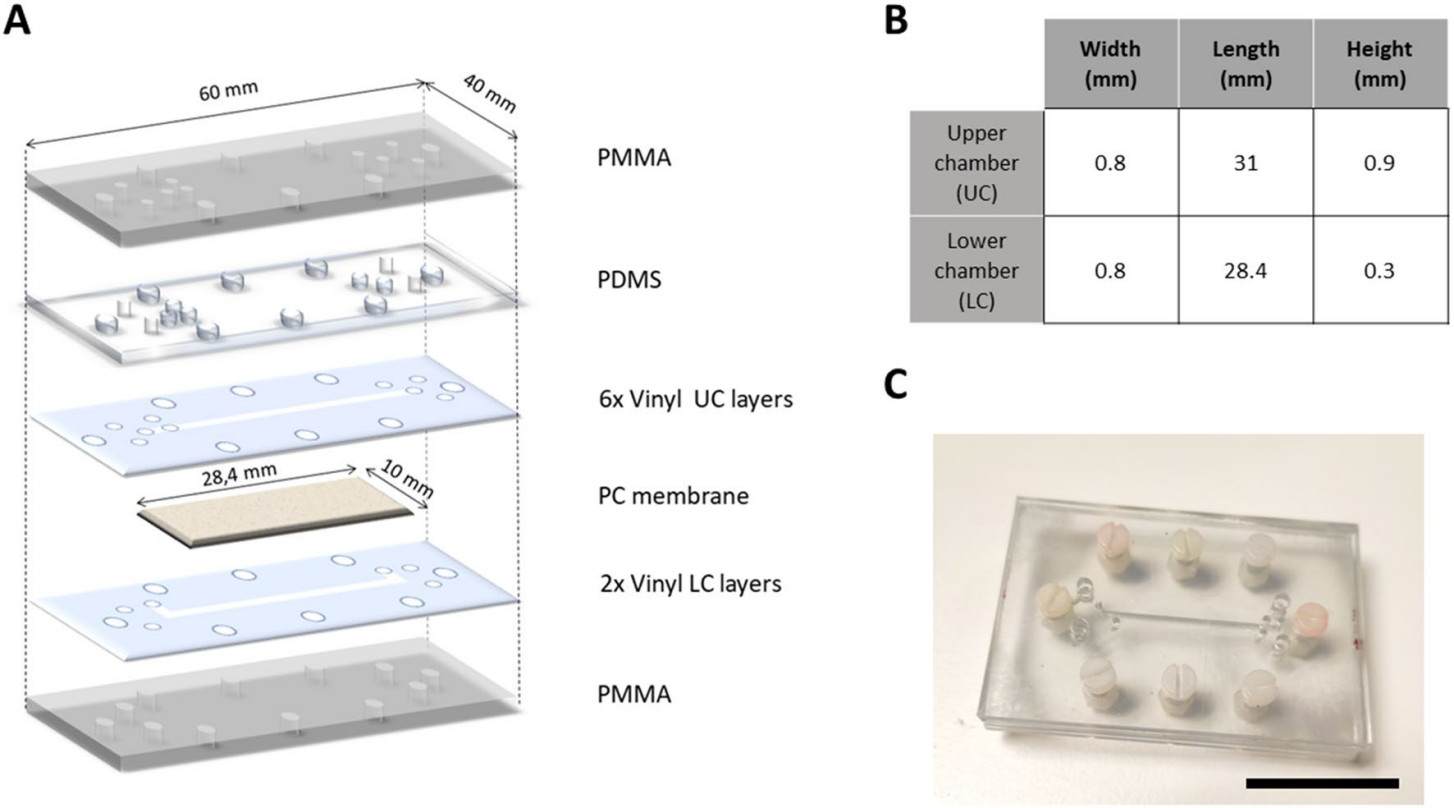
(**A**) Assembly of the chip with its different layers. (**B**) Table with the dimensions of the chambers of the chip. See also Fig. S1. (**C**) Real picture of the assembled chip. Scale bar: 3 mm.

In contrast to lithography-based chips, the cut and assemble manufacturing method used only requires an edge plotter (Summa S2 plotter), adhesive vinyl tape (Mactac MACal® 9800 PRO), and methacrylate sheets. The chip layers were designed in CAD software (FreeCAD version 0.18) and cut on the plotter. The assembly of the layers is carried out by means of an aligner tool fabricated ad hoc, which ensures the correct alignment of the consecutive layers that compose the complete stack (Fig. 2A). For this purpose, the chip design includes four 2.54 mm diameter holes in the vinyl and methacrylate layers matching the guides of the aligner tool (Fig. 2C). Figure 2B shows the dimensions of the chip chambers used as proof of concept in this article. The vinyl plotter provides a cutting resolution of 12.7 µm, which is enough for our application needs, considering that a typical cell size is 25–30 µm. Occasionally, we visualize (see Fig. S6) some defects produced during the assembly of the layers and the alignment of PDMS holes. Its size is up to 50 µm. These defects do not affect the proper operation of the chip.

The complete architecture of this chip comprises (Fig. 2A) 2 vinyl layers for the LC; a PC membrane covering the LC; 6 vinyl layers for the UC and a 500 µm-thick PDMS sheet (Sylgard Dow Corning) placed on top of the vinyl layers for two purposes: sealing the UC and providing appropriate anchor to the tubes that connect the chip to the pumps (see “Steps for the generation of the bilayer tissue model inside the chip” and Fig. 2C). The last vinyl layer is a double-sided adhesive sheet used to prevent any leakage from the chamber. Finally, the chip is fastened with eight nylon screws and nuts between two methacrylate (PMMA) rigid plates to make a monolithic sandwich structure that keeps the chip perfectly flat, provides a robust structure facilitating its handling, and assures water tightness (Figs. 2C and 4C). Both fluidic chambers are accessed through 1.2 mm diameter holes at the topmost PMMA plate where the inlet and outlet tubes are inserted (Fig. 2). Supplementary Video S2 shows the assembly process.

### Generation of the bilayer tissue model

#### Parallel flow model

To generate the stromal compartment inside the device’s upper chamber with a determined and constant height, we designed a parallel flow system between two liquids (pregel and PBS). Initially, we failed to generate a uniform layer loading only the pre-gel solution. This is a critical step since, upon gelation, the pregel will give rise to the stromal compartment of the bilayer tissue model.

Parallel flow of two fluids takes place at low flow rates in horizontal channels where a fraction of the channel is occupied by a fluid phase. They have a well-defined interface, and their viscosities largely affect the velocity^54^. To solve this parallel flow problem, we used the Navier–Stokes equations^55^ and continuity equation [Eq. (1)] with their corresponding boundary conditions. To solve the equations, we used the viscosity measurements obtained in the rheological tests described in “Study of the injectability”.

1 $$\begin{gathered} \frac{{\partial { }v_{x} }}{\partial x} + \frac{{\partial { }v_{y} }}{\partial y} = 0 \hfill \\ \frac{{\partial \left( {\rho v_{y} } \right)}}{\partial t} + \frac{{\partial \left( {\rho v_{x} v_{y} } \right)}}{\partial x} + \frac{{\partial \left( {\rho v_{y}^{2} } \right)}}{\partial y} = - \frac{{\partial { }p}}{\partial y} + {\upmu }\frac{{\partial^{2} { }v_{y} }}{{\partial y^{2} }} \hfill \\ \frac{{\partial \left( {\rho v_{x} } \right)}}{\partial t} + \frac{{\partial \left( {\rho v_{x}^{2} } \right)}}{\partial x} + \frac{{\partial \left( {\rho v_{x} v_{y} } \right)}}{\partial y} = - \frac{{\partial { }p}}{\partial x} + {\upmu }\frac{{\partial^{2} { }v_{x} }}{{\partial x^{2} }} \hfill \\ \end{gathered}$$

**Figure 3:**
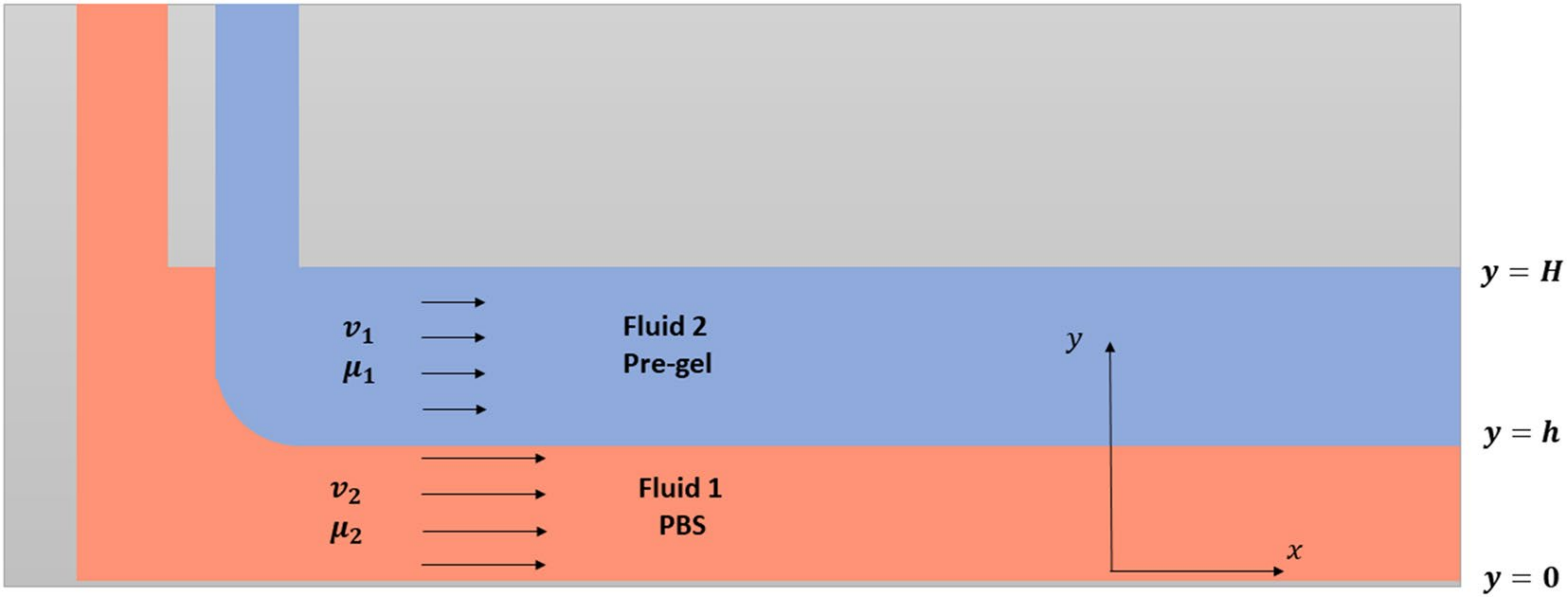
Scheme of the parallel flow model. Fluid 1, flowing on top, is the sacrificial fluid, PBS. Fluid 2 is the pre-gel solution containing the fibroblasts. The value of h changes as a function of the flows.

Fluid 1 corresponds to a sacrificial fluid, in our case PBS (Fig. 3), which has fluidic properties similar to those of water (1 cP). Fluid 2 is the pregel containing the hFBs, which is a non-Newtonian fluid (Fig. 3). We used its dynamic viscosity obtained by rheology (20 cP; see “Study of the injectability” and Fig. 6).

2 $$\begin{gathered} Q_{1} = w \left( { - \frac{{P_{l} }}{{6\mu_{1} }} \left( {H^{3} - h^{3} } \right) + \frac{{A_{1} }}{2}\left( {H^{2} - h^{2} } \right) + B_{1} \left( {H - h} \right)} \right) \hfill \\ Q_{2} = w \left( { - \frac{{P_{l} }}{{6\mu_{2} }} h^{3} - \frac{{A_{2} }}{{2\mu_{2} }}h^{2} + \frac{{B_{2} }}{{\mu_{2} }}h} \right) \hfill \\ \end{gathered}$$

${\mathrm{A}}_{1},{\mathrm{A}}_{2},{\mathrm{B}}_{1},{\mathrm{B}}_{2}$ are the integration constants, and ${\mathrm{P}}_{\mathrm{l}}$ is the pressure gradient along the channel. These [Eq. (2)] are subjected to boundary conditions:

- No-slip conditions at the walls:
  At $$y=0\to {v}_{2}=0$$
  At $$y=H\to {v}_{1}=0$$
- At the interface ($y=h$) we impose:
  Continuity of velocity: ${v}_{1}={v}_{2}$
  Continuity of shear stress: ${\mu }_{1}\frac{\partial {v}_{1}}{\partial y}= {\mu }_{2}\frac{\partial {v}_{2}}{\partial y}$

By imposing these boundary conditions and the desired height of the hydrogel, the equations can be solved, providing combinations of flow rates that lead to that height.

In the case of the proposed dermo-epidermal model, H (the height of the UC) is 900 µm, h (the desired height of the dermal compartment within the UC) is 500 µm, ${\upmu }_{1}, {\upmu }_{2}$ are the viscosities of the two fluids (1 and 20 cP, respectively), and *w* (channel width) is 800 µm.

#### Steps for the generation of the bilayer tissue model inside the chip

**Figure 4:**
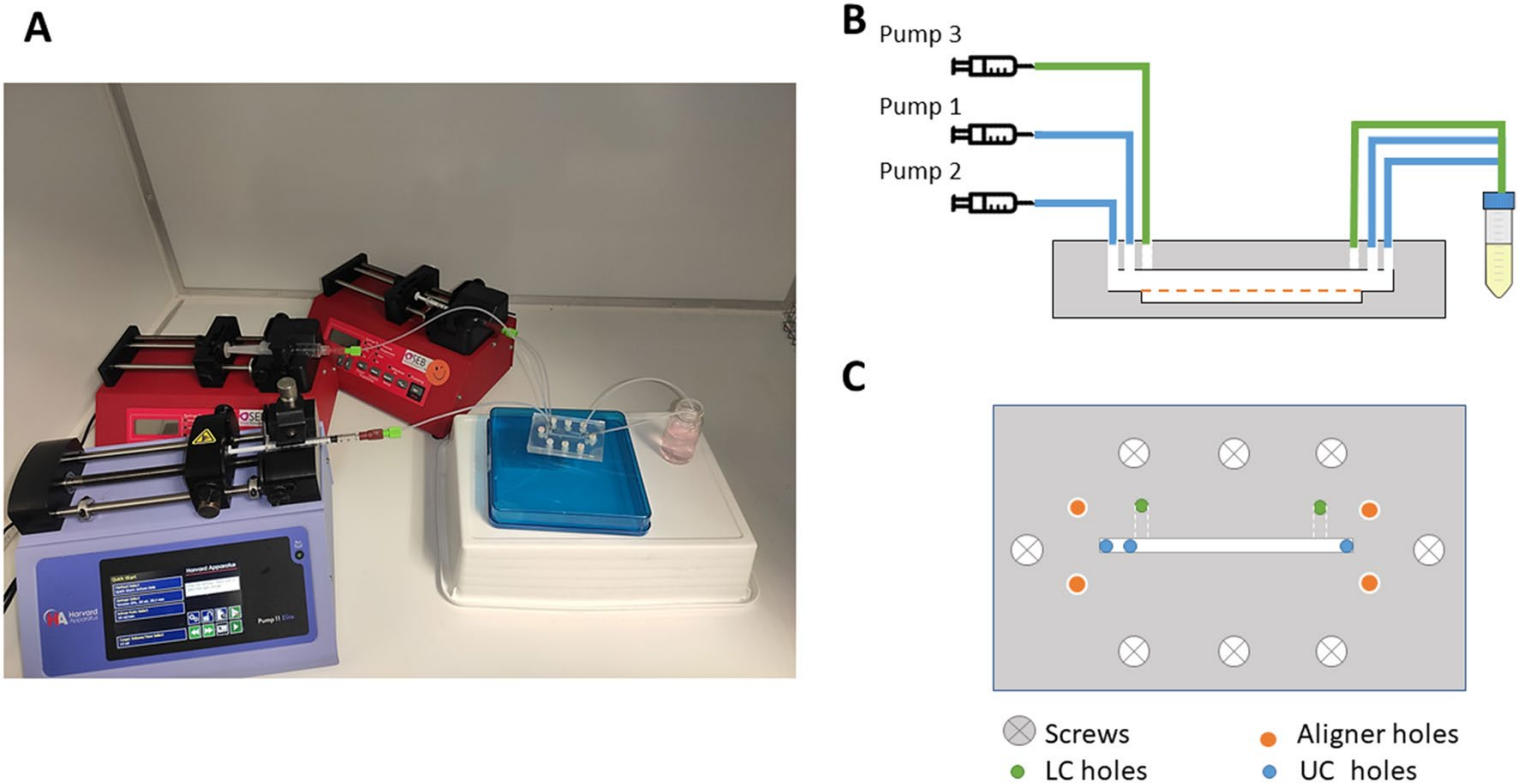
(**A**) Picture of the set-up. (**B**) Cross-section scheme of the chip and its connections to the pumps. Tubes for LC (green) and UC (blue) inlets and outlets. (**C**) Close top-view of the chip with tube connections.

Prior to the experiment, the chip was sterilized. UV light was used for at least 15 min to sterilize the PDMS and PMMA sheets before their assembly. Then, the chip is completely assembled in the laminar hood to preserve sterility. We flushed 70% ethanol for 5 min and then rinsed it with distilled water for another 5 min. Fluid flows were established and controlled by independent syringe pumps (PHD-Ultra Harvard Apparatus) that were connected through Teflon tubes (Tubing Natural 1/16"OD × 0.20" ID × 50 ft, IDEX) to the inlet holes of the chip in the PMMA upper sheet (Fig. 4).

**Figure 5:**
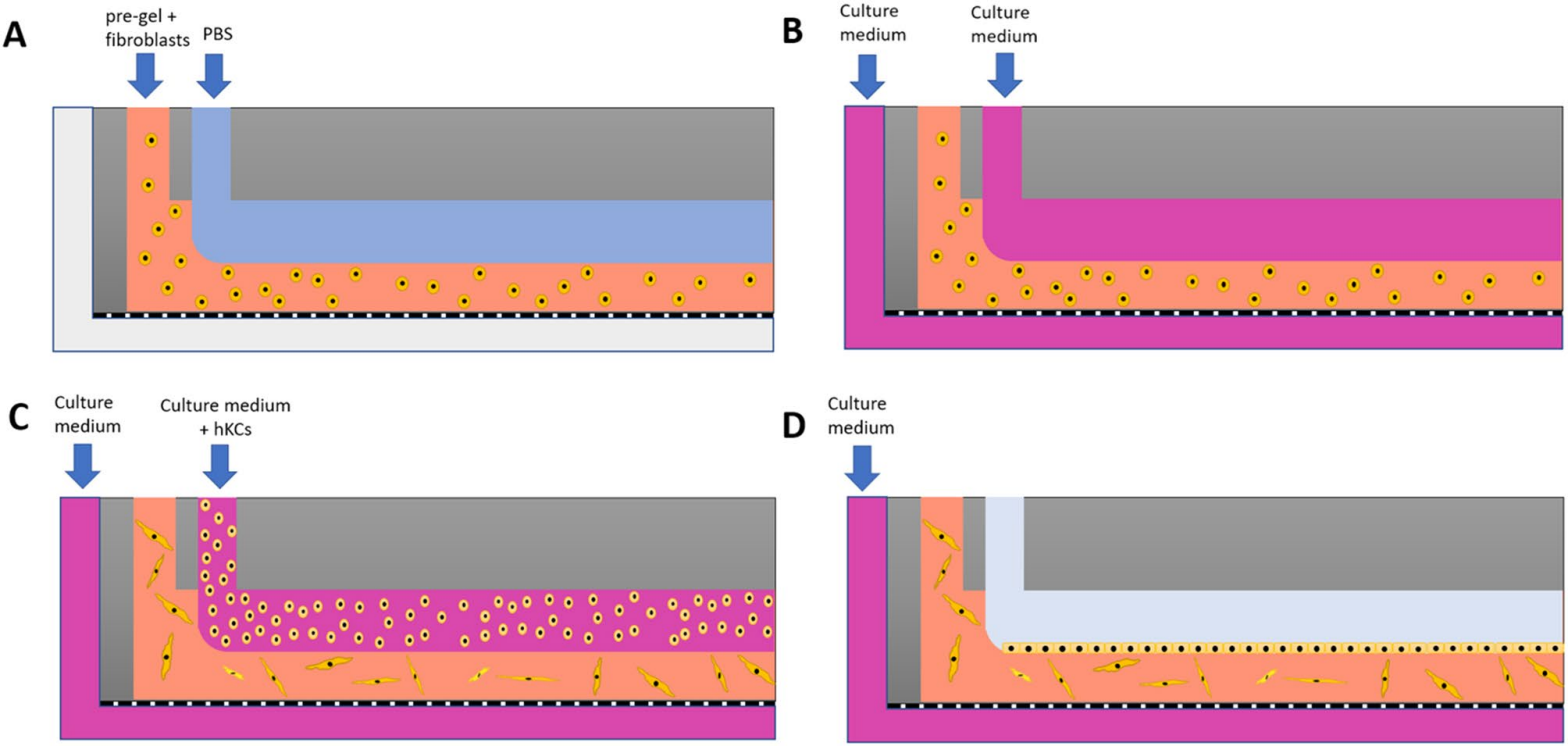
Steps followed to generate a bilayer tissue model inside the microfluidic device. (**A**) Parallel flow of PBS and pre-gel with hFBs. (**B**) Supplemented culture medium is pumped for 24 h to nurture the hFBs. (**C**) DMEM containing hKCs is pumped on top of the dermal compartment and kept resting for 6 h to allow cell attachment to the fibrin hydrogel surface. (**D**) As a final step, DMEM should be removed from the UC by pumping air to leave the hKCs exposed to the air–liquid interface to induce their differentiation and stratification. Grey colour represents air in the UC.

Syringe 1 (Terumo, 1 mL) totally filled the upper chamber with PBS at 100 µL/min and was then kept running at the same speed. A 500 µL fibrin pregel solution containing hFBs (previously described in “Study of the injectability”) was loaded into syringe 2 and run at 200 µL/min, displacing the lower part of the sacrificial PBS layer and forming a parallel flow with PBS (Figs. 4B and 5A). Once the pregel exited the upper chamber through the outlet tubes, both pumps were stopped. The chip was kept at 37 °C for at least 10 min for the pregel to gelify and generate the dermal compartment of the tissue. Then, supplemented culture medium was pumped at a rate of 40 µL/h through the lower chamber (by pump 3) throughout the rest of the experiment. Similarly, culture medium was also pumped by pump 1 under the same conditions for 24 h to displace the PBS layer of the upper chamber and to allow the hFBs to spread while preventing the gel from drying (Fig. 5B). Finally, hKCs suspended in supplemented culture medium were loaded by pumping (pump 1) at a density of 10^7^ cells/mL on top of the stromal compartment at 50 µL/min for 1 min (Fig. 5C). Cells were allowed to attach for 6 h (Fig. 5D). Pumping culture medium through the lower chamber (Fig. 5D) supplies nutrients to the cells by diffusion through the porous membrane.

To ensure cell viability, the experiments described in “Study of the injectability” to “Cell viability assay” were performed at 37 °C in a humidity-saturated chamber. Since fresh culture medium is continuously perfused through the LC, it is not necessary to provide additional CO_2_. If required, (e.g., to induce terminal differentiation of the hKCs, to introduce a drug to be tested, to analyse the effects of a microbiome, to simulate the effects of a tear, or to have a circulating air flow) an empty space (pale blue in Fig. 5D) can be created on top of the tissue by pumping out the supernatant in the UC by means of an air flow by pump 1.

To demonstrate the possibility of generating a three-layer construct using a syringe pump, we pumped the LC with HaCaT-RFP cells at a density of 10^7^ cells/mL at 50 µL/min for 1 min. They were allowed to settle and attach to the membrane for at least 6 h with the chip placed upside down. Then, we turned around the chip and followed the methodology described in “Steps for the generation of the bilayer tissue model inside the chip” to generate the dermal and epidermal compartments in the UC using the parallel flow method.

#### Gel height estimation

To verify that the proposed parallel flow protocol was capable of generating a bilayered construct inside of the chip and that the experimental parameters provided by the mathematical model (see “Parallel flow model”) were appropriate, the height of the fibrin hydrogel was measured. To this end, a hydrogel was formed inside the chip, as explained in “Steps for the generation of the bilayer tissue model inside the chip” and Fig. 5, but without hFBs. After gelation, the upper PMMA and PDMS layers of the chip were removed, and 50 µL of GFP-producing hKCs in culture medium were seeded on top at a density of 5 × 10^6^ cells/mL with a micropipette. The device was immediately observed under a confocal microscope, and a z-stack was acquired starting at the porous membrane and finishing at the fluorescent hKC cell layer settled on top of the hydrogel. This experiment was repeated three times with similar results.

### Cell viability assay

Cell viability in the microfluidic chip was assessed by staining hFBs and hKCs with a Live/Dead Viability/Cytotoxicity Kit for mammalian cells (Invitrogen) in separate experiments. For hFB viability, twenty-four hours after generation of the fibrin hydrogel, as described in “Steps for the generation of the bilayer tissue model inside the chip”, the culture medium was replaced with PBS in the UC and LC. After 5 min, air was pumped through pump 1 to remove PBS from the UC at a rate of 50 µL/min for 2 min. Then, 0.5 µL of 4 mM calcein-AM and 2 µL of 4 mM ethidium homodimer in PBS were pumped (pump 1) at 50 µL/min until the channel was completely filled. The chips were kept for 30 min at 37 °C in the dark in a cell incubator. Then, they were washed by pumping PBS at 50 µL/min for 2 min and observed under a confocal microscope (see “Fluorescence microscopy image acquisition”).

For hKC viability assays, a fibrin hydrogel without hFBs was formed, and hKCs were seeded on top of it and allowed to attach following the protocol described in “Steps for the generation of the bilayer tissue model inside the chip”. Then, the live/dead staining was performed as explained for hFBs. In this assay, green fluorescence denotes live cells, and red fluorescence denotes dead cells. These experiments were performed twice with very similar results.

### Fluorescence microscopy image acquisition

To reconstruct a 3D image of the tissue in the chip, confocal microscopy of a dermo-epidermal construct containing GFP-labelled hFBs and HKCs was carried out with a Zeiss LSM 710 confocal microscope. Z-stack images were obtained with a 10 × magnification objective and a z step of 10 µm. z = 0 was set at the porous membrane height as a reference, and slices were captured until no fluorescence was observed. Excitation and emission wavelengths of 495 and 519 nm, respectively, were used.

In the viability tests, images of green fluorescence were acquired using excitation and emission wavelengths of 495 and 519 nm, respectively. Red fluorescence images were obtained using excitation and emission wavelengths of 590 and 617 nm, respectively. Maximum intensity projections were generated from z-stacks using Zeiss software.

Time-lapse video (Supplementary Material) was obtained by fluorescence microscopy using a Leica Dmi8 inverted microscope equipped with an OKOLab incubator.

## Results

A critical problem we found when trying to generate a bilayer skin construct in the chip was the impossibility of finding appropriate injection conditions allowing the reproducible formation of a dermal compartment of homogeneous height along the UC, on top of which epidermal cells were deposited. To overcome this problem, we designed a parallel flow method based on the generation of two superimposed laminar flows, the lower one being the dermal pregel, while the upper one was sacrificial PBS (see “Steps for the generation of the bilayer tissue model inside the chip” and Fig. 5A). Consequently, we had to mathematically model this system to find the appropriate flow rates to obtain the desired dermal compartment height [see Eqs. (1) and (2), “Parallel flow model”].

**Figure 6:**
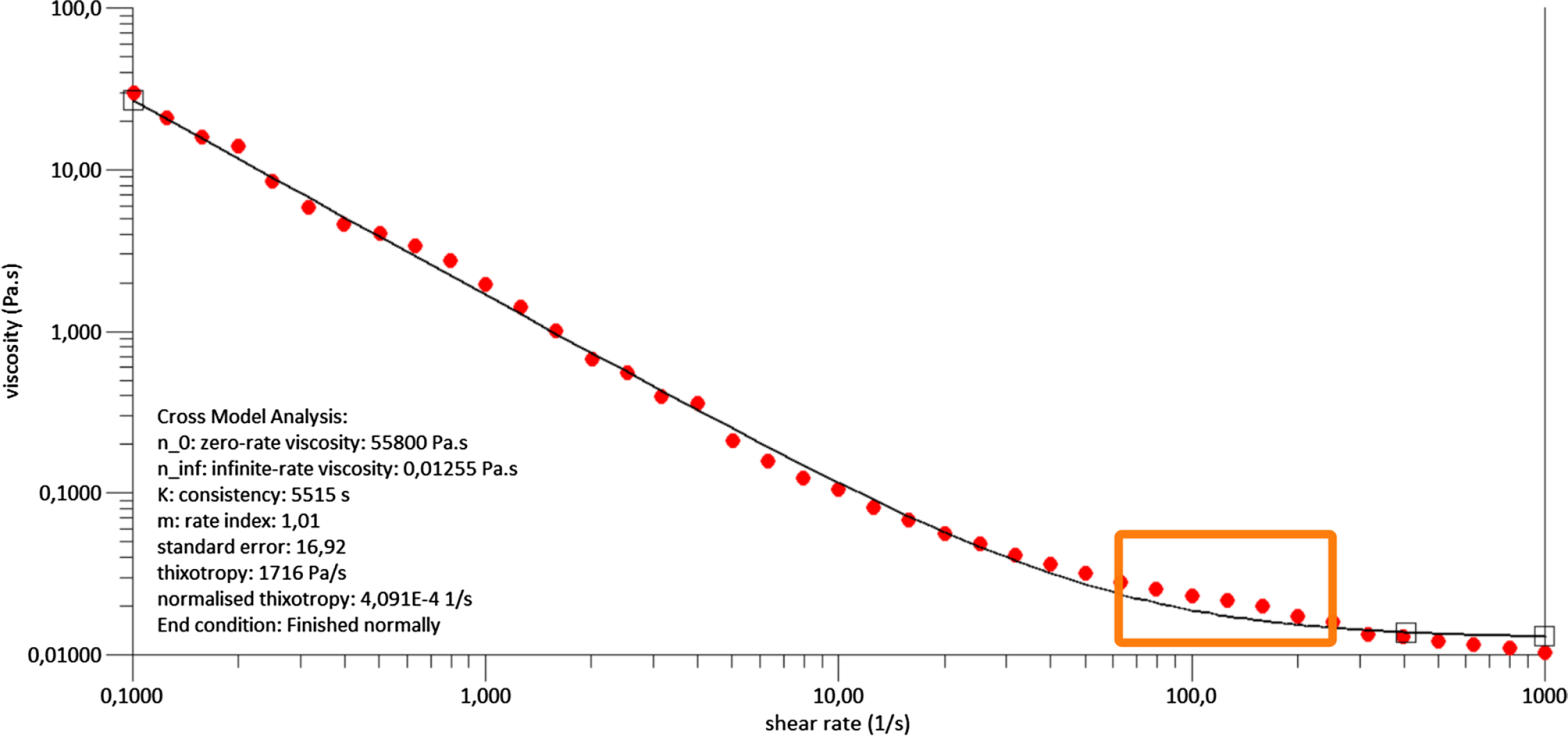
Viscosity vs. shear rate values obtained from rheological experiments. Representative dataset obtained in the rheometer for K and m estimation using the Cross Model. Note the good agreement between the red experimental data and the black line obtained from the model. The orange square denotes the shear rate range corresponding to the flow rates (50–200 µL/min) determined experimentally as the appropriate working conditions.

The solution of these equations requires the values of the viscosities µ_1_ and µ_2_ of the two liquids. The sacrificial fluid (PBS) viscosity µ_1_ was considered the same as that of water (1 cP). The viscosity of the fibrin hydrogel (pregel) was determined experimentally by rheology in the shear rate range to which the fluid was subjected to the tubing (Fig. 6).

Preliminary attempts in our laboratory showed that our fibrin pre-gels had a polymerization time (60–90 s) that was too short under static conditions once deposited in the UC. For this reason, the flow rate had to be as high as possible to avoid polymerization inside the tubes but not too high to avoid cell damage due to shear stress. After several tests, we obtained acceptable flow rates ranging from 50 to 200 µL/min. Introducing these flow rates (Q) and a value of 254 µm for the inner radius (R) of the Teflon tubes in Eq. 3^46^, the shear rate ($\dot{\gamma }$) varies from 64 to 260 s^−1^ (Eq. 3).

3 $${\dot{\gamma }} = { }\frac{{8{\text{Q}}}}{{2{\pi R}^{3} }}$$

Figure 6 presents the rheological data of viscosity vs. shear rate for the dermal pregel. The orange square in Fig. 6 shows the viscosity range corresponding to the shear rate range determined above (64–260 s^−1^). We will use the average viscosity value of 0.02 Pa s or 20 cP as µ_2_ to calculate the values of the flow rates for the parallel flow. Additional datasets illustrating a similar pre-gel rheological behaviour are shown in the Supplementary Material.

In addition, we observed that the viscosity presents a linear variation in the considered shear rate range, which is characteristic of shear thinning behaviour. This was confirmed by applying the Cross Model Equation [Eq. (4)]^56–58^:

4 $$\frac{{{\upeta } - {{ \upeta }}_{\infty } }}{{{\upeta }_{0} - {\upeta }_{\infty } }}{ } = { }\frac{1}{{1 + \left( {{{{\text{K}}\dot{\gamma }}}} \right)^{{\text{m}}} }}$$

${\eta }_{\infty }$ and ${\eta }_{0}$ are the viscosities corresponding to the lower and higher shear rate values, respectively, of the shear range studied (64 and 260 s^−1^), and $K$ is the consistency (5515 s). $\mathrm{m}$ is an index that indicates the degree of shear thinning behaviour of the pre-gel. If it takes values close to zero, the fluid presents Newtonian behaviour, and if $m$ is close to one, the fluid presents shear thinning. In our case, $\mathrm{m}=1.01$ under our working conditions. This shear thinning behaviour is the reason the hydrogel flows under these working conditions, while in static conditions, it polymerizes faster.

**Figure 7:**
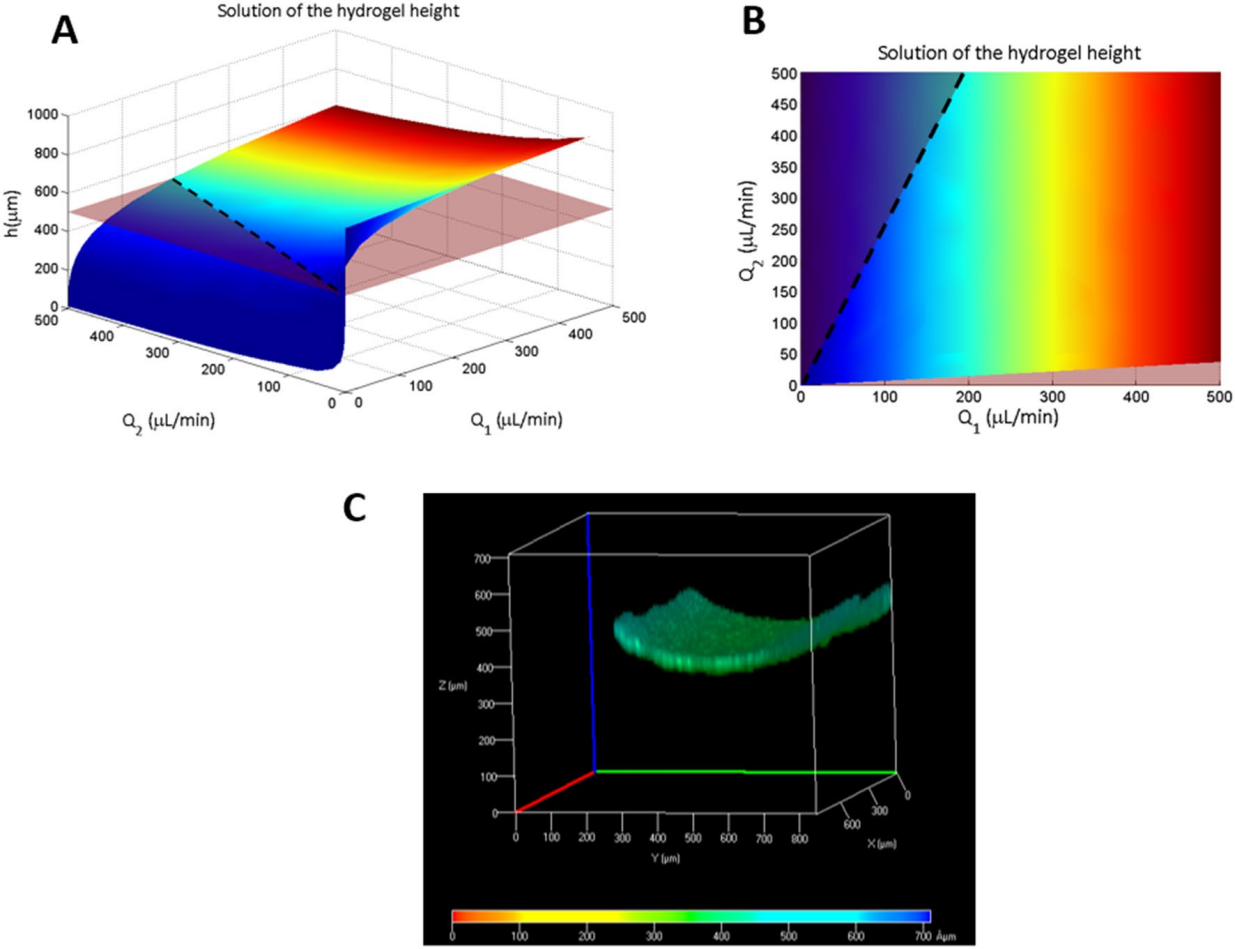
(**A**) Surface of flows (Q_1_ for the sacrificial fluid and Q_2_ for the pre-gel) as a function of the height predicted by the parallel flow mathematical model. The plane z = 500 µm plotted in pink corresponds to the height chosen for the dermal compartment (hydrogel). The intersection line between the surface of solutions and z = 500 µm is depicted as a dashed black line. (**B**) Top view of the solution in (**A**) showing the dashed black line of possible flows (Q_1_ and Q_2_) to obtain a dermal compartment 500 µm high. (**C**) Confocal image of the gel. The Y axis correspond to the width of the chamber and the X axis correspond to the length of the chamber. The Z axis represents the height of the chamber. z = 0 corresponds to the position of the porous membrane. The figure represents a field of view of 800 µm × 800 µm in the XY plane at the centre of the UC. The colour code at the bottom of the figure indicates the height at which the seeded fluorescent hKCs are found (the hydrogel’s surface).

Now numerically solving Eq. (2) leads to the surface of possible solutions for both flow rates (Q_1_ and Q_2_) as a function of the height of the hydrogel. In our case, setting the hydrogel’s height at h = 500 µm (pink plane in Fig. 7A), the intersection of this plane with the surface of solutions defines the line of possible flow rates to obtain that height (dashed black line in Figs. 7A,B). In practice, we used Q_1_ = 100 µL/min and Q_2_ = 200 µL/min, leading to a theoretical hydrogel height of 525.4 µm.

To experimentally check the predictions of the method, the height of the hydrogel along the upper chamber was measured (Fig. 7C) as described in “Gel height estimation” of the “Materials and methods”, and a slightly concave surface of the hydrogel was visualized. This U shape could probably be due to wall effects. Spite of this, an average height of 475 µm was observed (Fig. 7C), in good agreement with the prediction of our mathematical model.

To demonstrate that the described model worked correctly, we included other similar experiments carried out with three different combinations of flow rates in the Supplementary Material. In the three cases (Figs. S3, S4, and S5), the difference between the experimental values and the ones theoretically set for height was $\le 8.3 \%$, showing the versatility of the parallel flow method and the usefulness of the mathematical model predicting the appropriate flow rates. These results also demonstrate that, despite their complexity, these experiments are reproducible.

**Figure 8:**
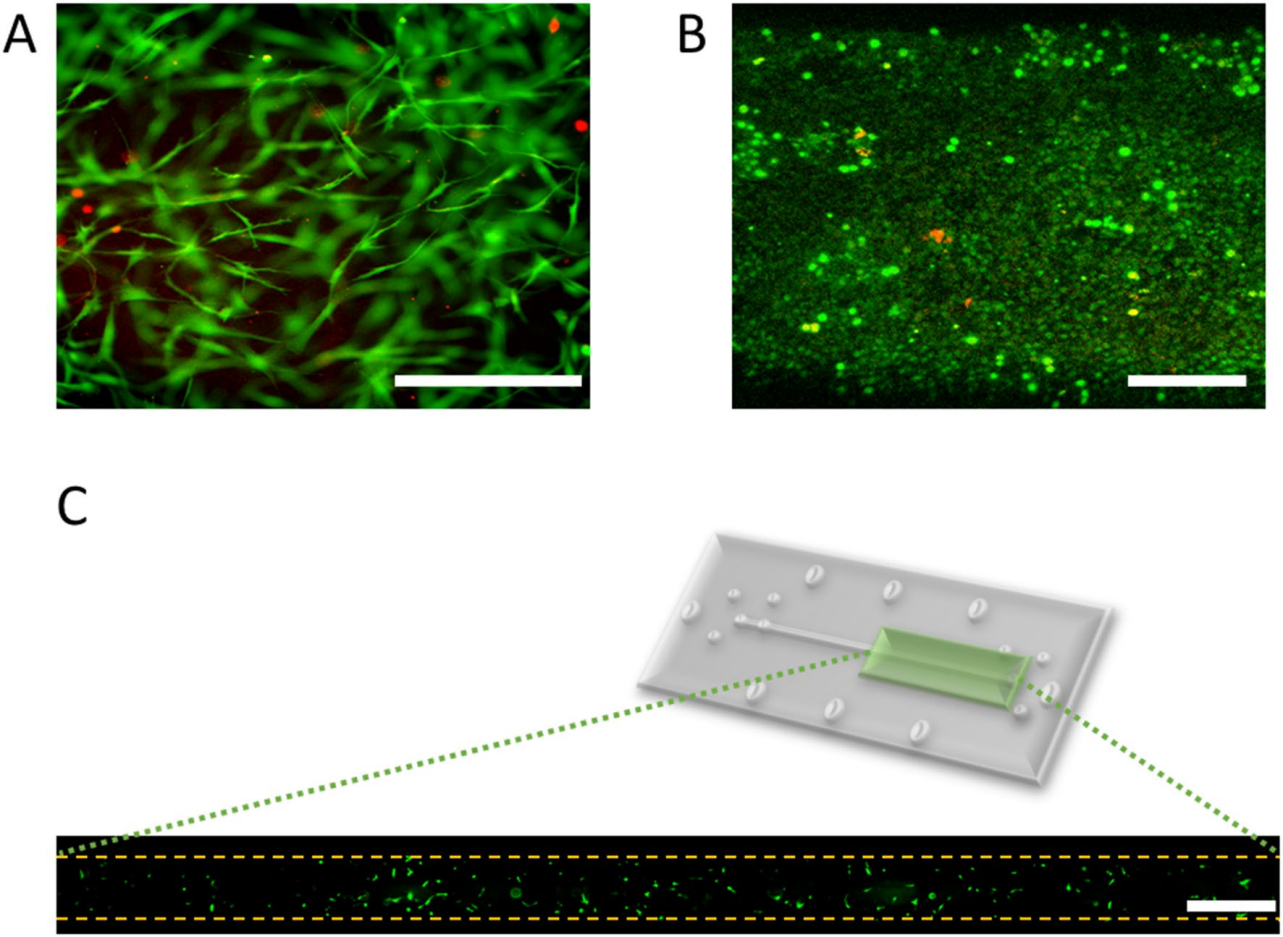
Live/Dead assay in the microfluidic chip. Green and red fluorescence denote live and dead cell, respectively (**A**) hFBs inside of the fibrin hydrogel 4 days after loading in the channel. Scale bar: 100 µm. (**B**) hKCs after 4 days after seeding on top of the fibrin hydrogel. Scale bar: 200 µm. The figures are maximum intensity projections from z-stacks obtained with confocal microscopy. (**C**) Top view of the UC 24 h after loading a fibrin hydrogel containing GFP-hFBs using the parallel flow method. The upper figure indicates the region of the UC that is presented enlarged in the lower figure. GFP-hFBs are homogeneously distributed along the chamber and display a spindle shape, indicating that they are well spread. Scale bar: 1000 µm.

We used the live/dead test to analyse to what extent the experimental process affects cell viability. Figure 8A shows a representative picture of the results obtained with hFBs embedded in the hydrogel for 4 days after loading using the parallel flow method. Analysis of ten fields demonstrated a cell viability of 98% $\pm$ 2%. Similar tests performed with hKCs 4 days after seeding them on top of a fibrin hydrogel showed a 99% $\pm$ 4% viable cells (Fig. 8B).

Figure 8C shows a fluorescent top view image of the upper chamber containing a fibrin hydrogel with embedded GFP-hFBs, demonstrating that 24 h after loading, cells are uniformly distributed along the chamber and spread well. Additionally, a time-lapse video was recorded to visualize the behaviour of the GFP-hFBs inside the hydrogel for 24 h, starting 24 h after loading. This video shows well spread and very motile cells (Supplementary Material Video S1). Collectively, these results demonstrate that the conditions established for the parallel flow method of loading do not significantly affect the viability of the two types of cells (Fig. 8).

**Figure 9:**
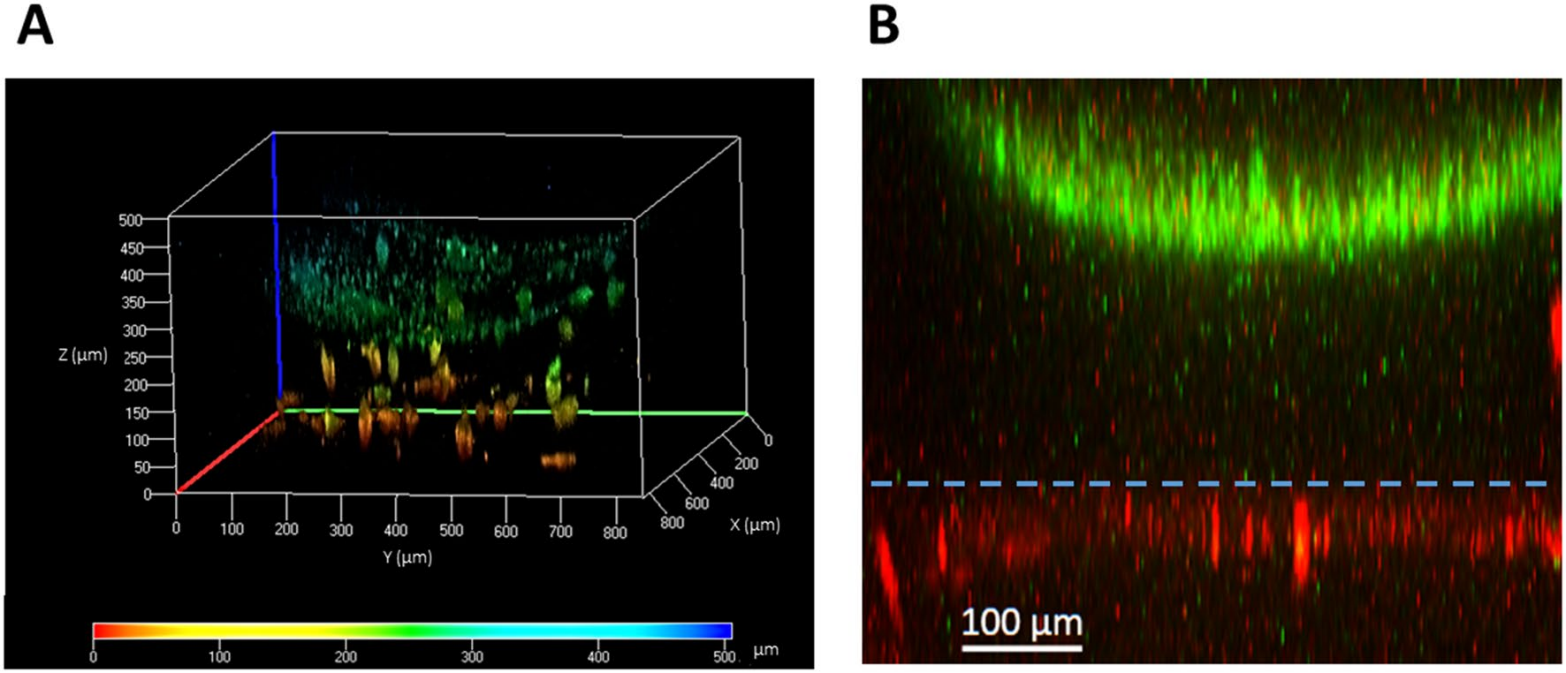
(**A**) Confocal image of a bilayer dermo-epidermal equivalent generated in the UC of the microfluidic device using the parallel flow method. The colour code indicates the height of the cells (Z axis). The lower, reddish objects correspond to hFBs and the green and smaller ones, on top of the hFBs, correspond to hKCs. (**B**) Confocal image of a three-layer construct composed of a layer of red fluorescent cells (HaCaT-RFP) seeded under the porous membrane (denoted with blue dashed line), an acellular fibrin hydrogel generated by parallel flow method and an epithelial layer composed of green-fluorescent cells (HaCaT-GFP) seeded on top of the hydrogel.

The next step was to generate complete dermo-epidermal constructs and study them using confocal microscopy (Fig. 9). These constructs were generated as described in “Steps for the generation of the bilayer tissue model inside the chip” of the “Materials and methods” using GFP-hFBS and GFP-hKCs. Twenty-four hours after seeding the hKCs on top of the hFB-containing dermal compartment and perfusing them with medium through the LC and UC, we found fluorescent cells both inside the hydrogel and on top of it, as expected (Fig. 9A). A colour code for height was used to better appreciate the three-dimensional aspects of the constructs obtained. The reddish cells, found at lower heights, correspond to hFBs, whereas the green cells are hKCs seeded on top of the hydrogel, which reproduced the characteristic U-shape and height reported in Fig. 7C with hydrogels without hFBs. Figure 9B shows the viability of hosting an additional layer in the lower chamber to mimic other tissues. As a proof of concept, we show HaCaT-RFP cells attached to the underside of the membrane that separates the UC from the LC.

## Discussion

We present a new type of microfluidic chip, alternative to traditional PDMS-based systems^59^, that is cost-effective, easy-to-use and produce, and is made of biocompatible adhesive vinyl sheets^44,45^. This system avoids the use of silicon wafers and complicated plasma bonding procedures needed in soft lithography with PDMS^42,43^. The only instrumentation required is a cheap vinyl plotter (300–400 €). The material and the micromachining method used here make ad hoc production of microfluidic chambers with different sizes and geometries very easy. Therefore, the method described in this article is simple, reliable and scalable to be implemented industrially.

In addition to its friendly characteristics and cost, this design is part of the current trend to seek substitutes for PDMS as a material for the manufacture of TOCs^44–46^. Despite recent improvements, PDMS presents limitations related to hydrophobicity and gas permeability, as well as the high cost of the facilities and instrumentation required and its still relatively low efficiency. A key element for the simplicity of our prototype is the use of biocompatible, pressure-sensitive adhesive vinyl sheets. The use of this type of material is one of the recently proposed alternatives to reduce microfluidic concept-to-chip time and costs. Adhesive tapes can be used to bond a large variety of industrially relevant polymeric materials, including some relevant materials for biochip construction, such as polymethylmethacrylate (PMMA), polycarbonate (PC), or polystyrene (PS).

As an inspiring example, as many adhesives are toxic to living cells and an appropriate interaction with the material is key for TOC systems, Kratz et al.^44^ characterized four biomedical grade adhesive tapes (three acrylic- and one silicone-based) for rapid prototyping, including structuring precision, physical and optical properties and biocompatibility. The four materials showed excellent optical properties, good manufacturability, low height tolerances and strong bonding to both glass and a variety of polymeric materials, such as membranes. The authors conclude that “adhesive tapes present a viable alternative to overcome the challenge of integrating multiple functional layers of different polymer types”.

Despite its positive aspects, our device suffers from two main drawbacks that need to be improved. The first is that it is limited to rectangular cross-sections. For our current research and many other applications, rectangular cross-sections are suitable, although this might not always be the case. However, with our method, it is possible to build more sophisticated chambers containing multiple compartments, as described in^60^, for gene synthesis in a microfluidic device. This would allow, for example, modelling and individually monitoring the interaction between various tissues or organs. The second limitation of our technique is the rigidity of the microfluidic structures, which limits their ability to deform in response to pressure changes or mechanical stress. Applying tension to the tissue can be achieved either by deforming the entire chip or just the membrane between the chambers, as demonstrated by Ingber et al.^28,37^ for cell monolayers using PDMS chips and membranes. A possible solution would be the use of elastomers, but the manufacturing methods required are much more complex than the very simple ones required with the materials of our method^46^, especially in regard to producing porous membranes. As an alternative, we are exploring methods that allow mechanical stress to be applied directly to tissues without the need to deform the structure of the device.

As mentioned in the introduction, to our knowledge, there is no description of any method allowing the generation of three-dimensional tissue models inside microfluidic chips. The main novelty of our work is the development of a method based on parallel flow to compartmentalize in different and homogeneous layers a three-dimensional structure, thus enabling the modelling of complex tissues, in particular epithelia, which are characteristically composed of a lower stromal and an upper epithelial component. This method differs from previously published methods^30,31,35–38,40,59^, which mainly model epithelial tissues as simple monolayers lacking a stroma. Therefore, our method allows better modelling of epithelial real structure and, consequently, the physiology and tumorigenesis of these tissues. There are other studies that include dermis and epidermis in their skin on a chip model, such as the work accomplished by Sriram et al.^18^. Nevertheless, they followed a manual seeding procedure which lacks the main advantages of the chip concept, although they obtained a well differentiated skin. Others^16,30,61^, however, took skin tissue (of commercial origin or from patient biopsies) and placed them in an insert adapted to the chip increasing their lifespan for testing purposes. For more details see reference^62^.

The parallel flow method establishes another main difference with respect to current technologies, namely, the loading process of the cellular components into the chips. In general, cells are manually pipetted into the culture channels, while our method allows the injection of all the components, including cells and extracellular matrices, directly into the channels using syringe pumps, thus allowing better automatization and standardization of the loading process.

We mathematically modelled the parallel flow system to obtain the flow rates Q_1_ and Q_2_ required to load the sacrificial PBS and the pregel, respectively, to obtain a stromal layer with a predetermined height (500 µm in our case) in the UC. This implied solving the Navier–Stokes equations, which in turn required the determination of the dynamic viscosity of the pregel by rheology. The experimental results matched fairly well with the theoretical values provided by the model, indicating that the method allows the modulation the height of the stroma according to the experimental needs. Moreover, the model provides different possible values for the pair Q_1_ and Q_2_, which adds flexibility to accommodate different experimental requirements and designs.

To show the versatility of the method and to validate the mathematical model, we performed additional experiments to demonstrate that different hydrogel heights of 300, 500 and 700 µm can be achieved by tuning the two-fluid flows (Figs. S3, S4, S5 in the Supplementary Material). In the three cases, the difference between the experimental value and the one theoretically set for height was $\le$ 8.3%, well within the experimental error of these experiments. Furthermore, the results of Figs. 7C and S7, 475 and 480 $\mathrm{\mu m}$, respectively, obtained using the same flows ${\mathrm{Q}}_{1}=100\mathrm{ \mu L}/\mathrm{min}$ and ${\mathrm{Q}}_{2}=200\mathrm{ \mu L}/\mathrm{min}$ predicted by the mathematical model to obtain a hydrogel height of 500 $\mathrm{\mu m}$ indicate that, despite their complexity, these experiments are reproducible.

Using skin as a model tissue, we first demonstrated that the parallel flow method did not affect significantly the viability of human fibroblasts and keratinocytes (Figs. 8 and S7), and then, we proceeded to generate 3D bilayer skin. To this end, we first loaded a fibroblast-containing fibrin pregel to give rise to the stroma of the tissue. Once the pregel polymerized, we seeded a layer of keratinocytes on top to simulate the epithelial compartment. The results obtained (Fig. 9) demonstrate that, using this method, it is possible to directly generate two-compartment (stroma and epithelium) 3D tissue in a microfluidic chip. We still lack more biological analysis of the modelled construct, such as increasing culturing times or performing immunocytochemistry to specific skin markers (keratins, filaggrin, vimentin, etc.) such as those provided by^18,30,39^.

The flexibility of the method allows us to leave void space at the top of the epithelium, which is the physiological situation of many tissues that line a void lumen (e.g., intestinal and urinary epithelia) or are in contact with the air (e.g., cornea, skin, oral and respiratory epithelia). This additional free space is needed to allow terminal differentiation of stratified epithelia (of which cornea, skin, and oral mucosa are paradigmatic examples), but it can also be used to introduce continuous or discontinuous flows of air, culture medium, drugs to be tested or even a microbiome^38,63,64^.

We observed that the hydrogel surface in the chip was not flat but U-shaped. We hypothesize that this effect is due to the expected interaction between the pregel and the vinyl walls (“wall effect”) and is magnified due to the submillimetric width (0.8 mm) of the channel^65^. In any case, this meniscus is not very pronounced and does not seem to significantly affect the functioning of the 3D tissue model or the parallel flow. There are different approaches to reduce this effect. One could be the treatment of the vinyl surface to change its hydrophobicity to minimize the contact angle between the pregel and the wall. Another possible solution would be increasing the channel width (for instance, to 2 mm, which will not disturb the laminar flow and therefore the parallel flow) to diminish the effect of the meniscus.

Regardless of these encouraging results, future work is needed to determine the long-term performance of the system and, in the case of epidermis, to verify the differentiation of the hKC monolayer to form a well-differentiated epidermis, including a stratum corneum, prior to using the system to test drugs.

## Supplementary Information

Supplementary Information 1.

Supplementary Video 1.

Supplementary Video 2.
