# Supplementary material for: A new microfluidic method enabling the generation of multi-layered tissues-on-chips using skin cells as a proof of concept
Source: Sci Rep. 2021 Jun 23;11:13160. doi: 10.1038/s41598-021-91875-z (PMC8222336; doi:10.1038/s41598-021-91875-z)
Supplement: Supplementary file 1 — Supplementary Information 1. [file 41598_2021_91875_MOESM1_ESM.docx]

**Supporting Information_ A new microfluidic method enabling the generation of multi-layered tissues on chips: A proof of concept using skin cells**

L. Valencia^a^, V. Canalejas^c^, M. Clemente^a^, I. Fernaud^e^, M. Holgado^c,d*^, J.L. Jorcano^a,b*^ and D. Velasco^a*^

^a^ Department of Bioengineering and Aerospace Engineering, Universidad Carlos III de Madrid (UC3M), Madrid, Spain

^b^Division of Epithelial Biomedicine, CIEMAT, Madrid, Spain
^c^ Group of Optics, Photonics and Biophotonics (GOFB). Center for Biomedical Technology. Universidad Politécnica de Madrid,Spain
^d^ Departamento de Física Aplicada e Ingeniería de Materiales, Escuela Técnica Superior de Ingenieros Industriales. Madrid, Spain

^e^ Laboratorio Cajal de Circuitos Corticales, Center for Biomedical Technology. Universidad Politécnica de Madrid. Campus de Montegancedo. Madrid. Spain

*Corresponding authors: E-mail: jjorcano@ing.uc3m.es, divelasc@ing.uc3m.es, m.holgado@upm.es

1. **Video fibroblasts time-lapse**

Video S1: A time-lapse video was also recorded to visualize the behaviour of the GFP-hFB cells inside the hydrogel for 24 hours, starting 24 hours after loading.

2. Video chip assembly

Video S2.

Recorded video showing chip assembly with two LC layers, PC membrane and two UC layers as an example. Custom-made aligner is shown. PDMS layer is added. Holes are done with a biopsy puncher. Then the chip is fastened with the methacrylate layers.

1. **CAD designs**


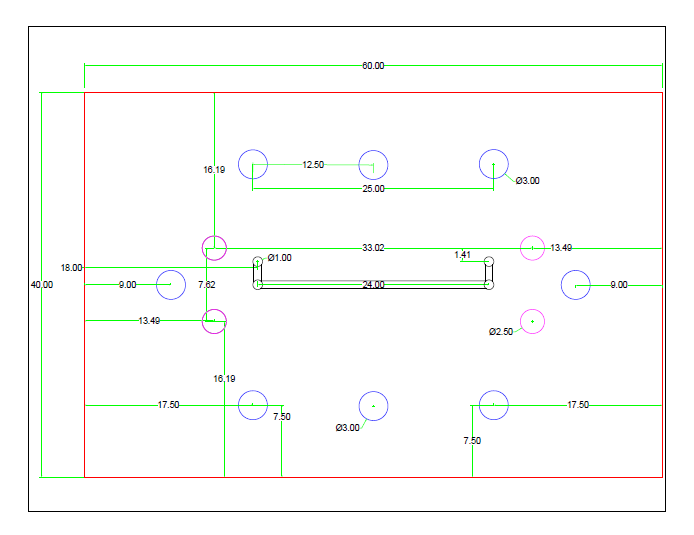

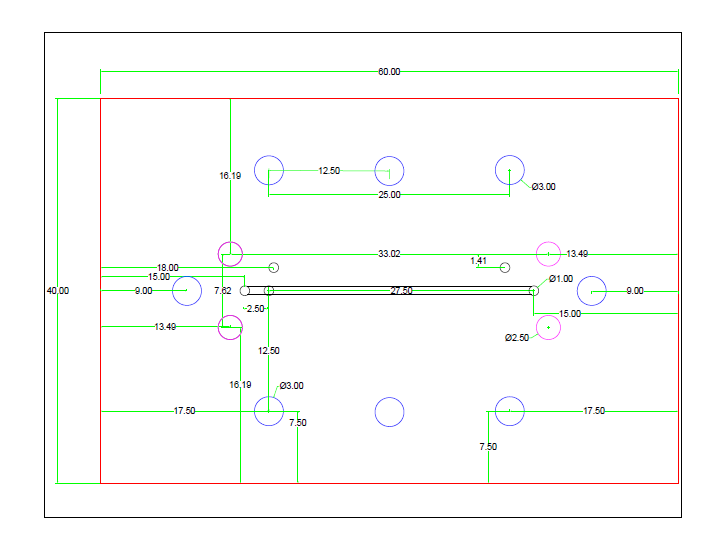


A

B

Figure S 1 CAD drawings for the A ) lower chamber (LC) and B) the upper chamber (UC). All channels are 800 µm-width. Screws holes are 3 mm diameter and aligner holes are 2.50 mm diameter. LC has a U-shape, so the inlets and outlets are easily identified. UC is a straight channel with two inlets for the parallel flow and a single outlet. Total size of the chip is 40x60 mm.

1. Rheology datasets.


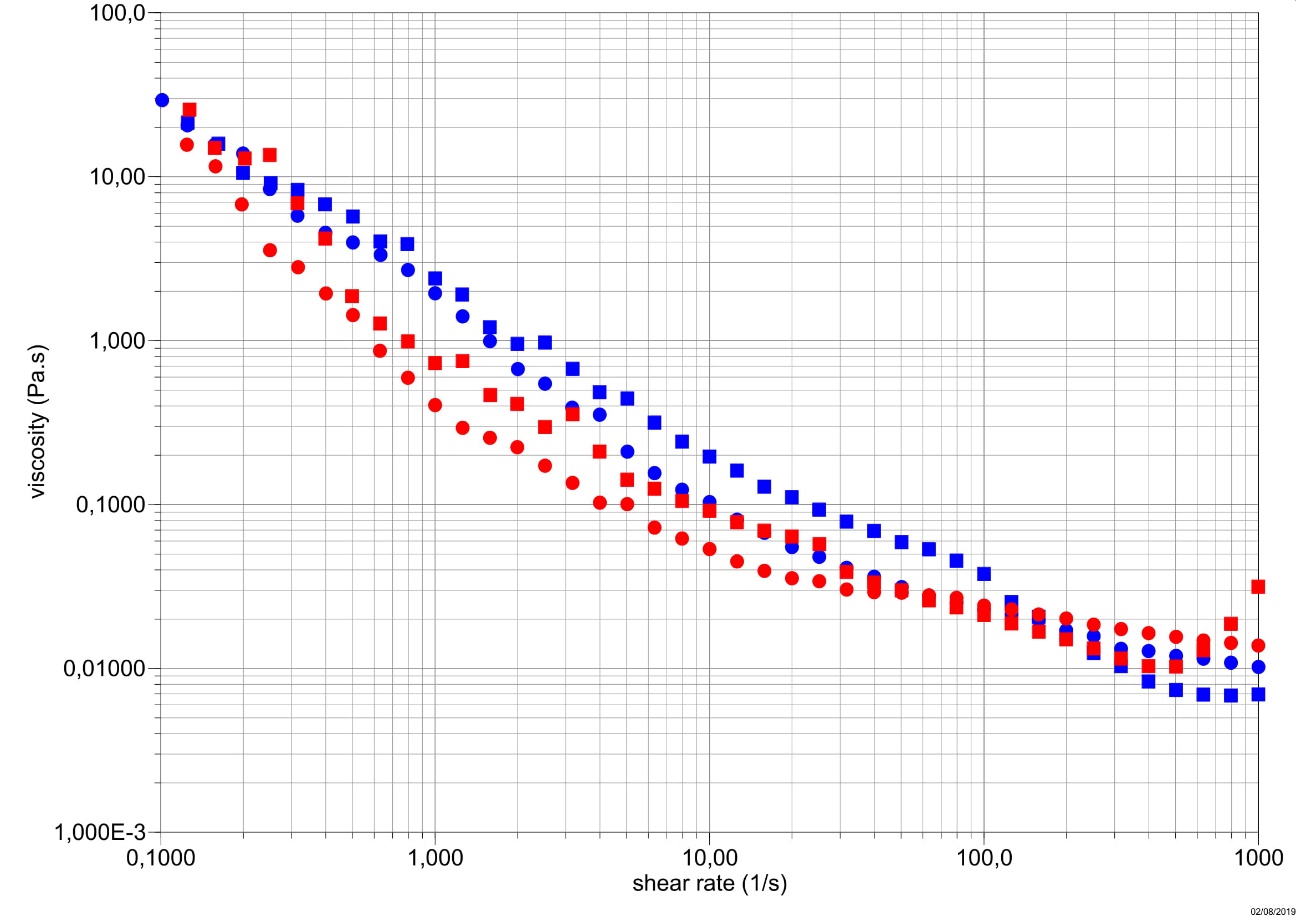


Figure S 2 Viscosity vs. shear rate values obtained from different rheological experiments.

1. Different hydrogel height obtained with parallel flow method.


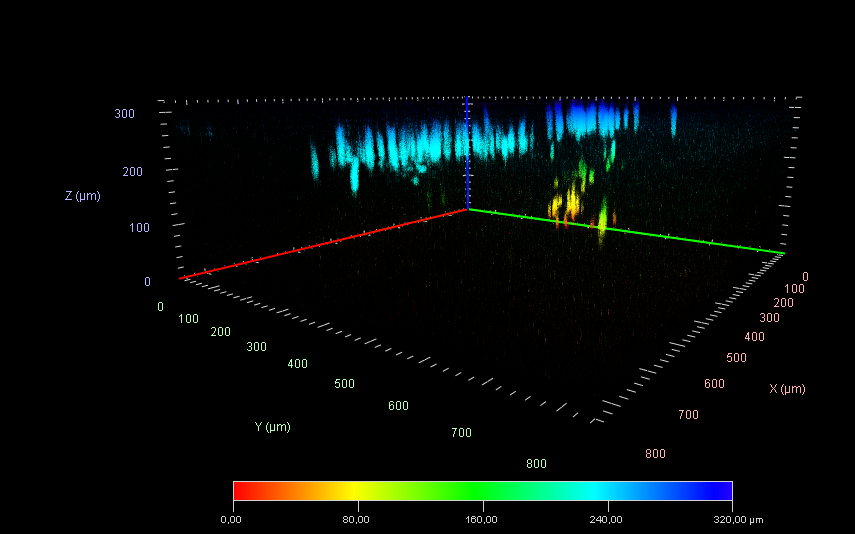


Figure S 3 Confocal microscopy of a fibrin hydrogel with HaCaT-h2B GFP seeded on top to measure the hydrogel’s height. Parallel flow performed at Q1=25 μL/min and Q2=350 μL/min. The average height of the hydrogel was 275 μm compared to the 300 μm set. Deviation= 8.3 %. Color coding indicates height of the sample for an easier interpretation.


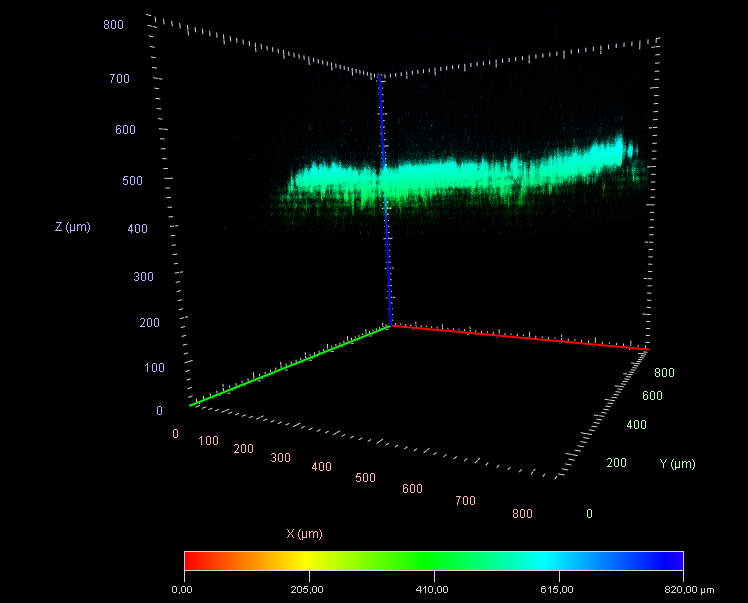


Figure S 4 Confocal microscopy of a fibrin hydrogel with HaCaT-h2B GFP seeded on top to measure the hydrogel’s height. Parallel flow performed at Q1=100 μL/min and Q2=200 μL/min. The average height of the hydrogel was 480 μm compared to the 500 μm set. Deviation= 4 %. Color coding indicates height of the sample for an easier interpretation.


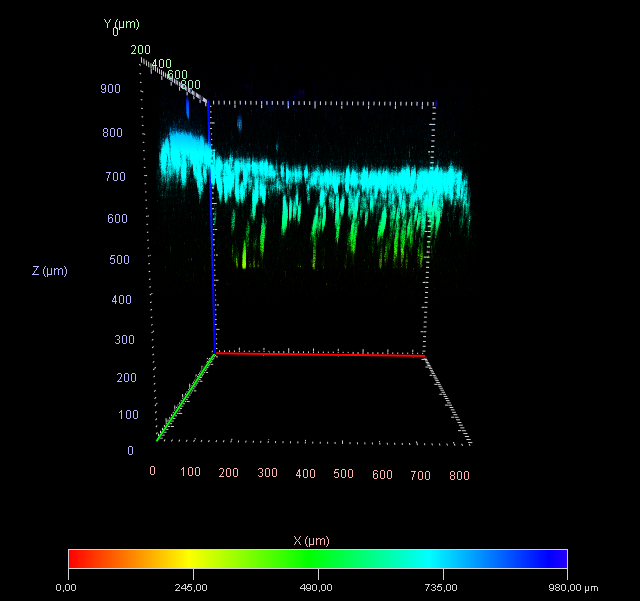


Figure S 5 Confocal microscopy of a fibrin hydrogel with HaCaT-h2B GFP seeded on top. Parallel flow performed at Q1=250 μL/min and Q2=100 μL/min. Averaged obtained height of the hydrogel was 660 μm compared to the 700 μm set. Deviation= 5.7 %. Color coding indicates height of the sample for an easier interpretation.

1. Cutting precision

The circles are marking the region with the most misalignment of the designed structures when piling up the consecutive layers and the PDMS holes performed with the biopsy punch. The maximum missalingment found is of 50 µm.


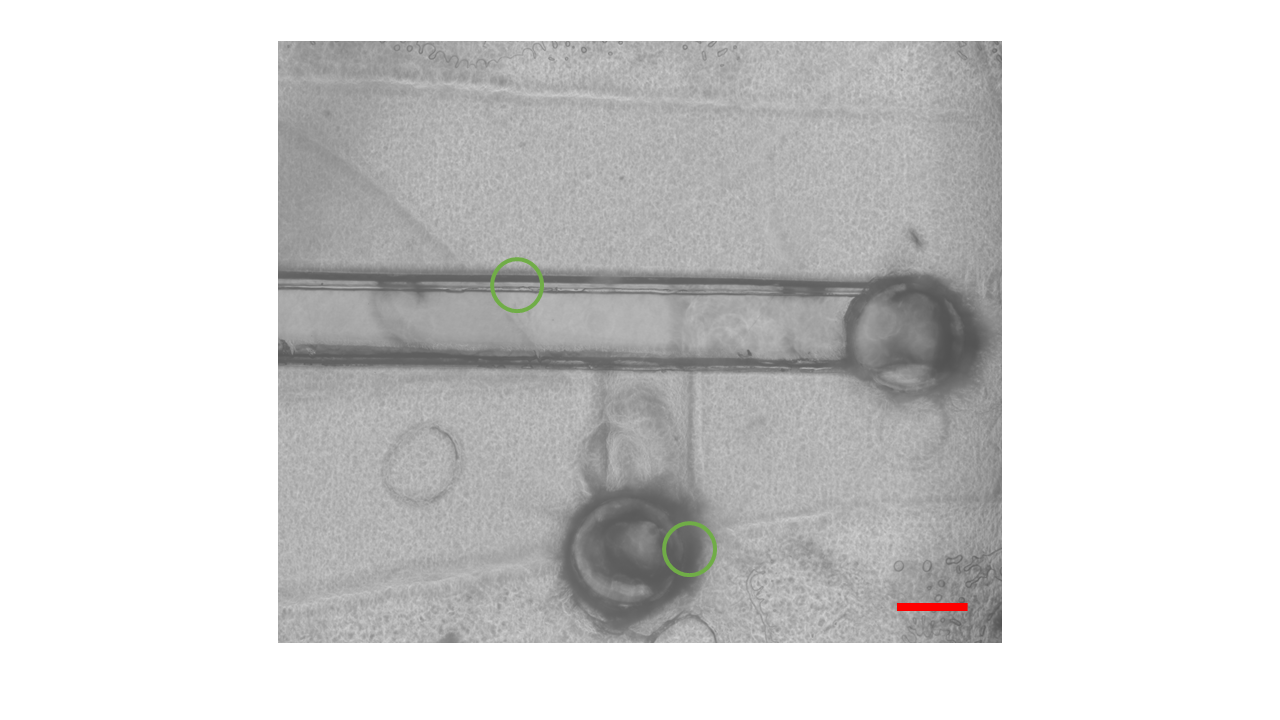


Figure S 6 Microscopy top view of the channel after chip assembly. Green circles denote defects on the assembly or due to precision of the edge plotter or PDMS layer assembly. Scalebar: 800 µm

1. Confluent monolayer seeding


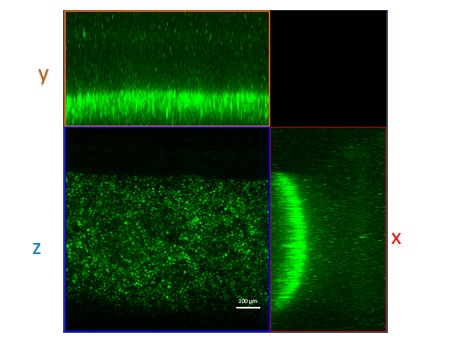


Figure S7 Orthogonal display with maximum intensity projection (MIP) from a z-stack confocal image. A cell monolayer of HaCaT-GFP was seeded on top of a hydrogel in the upper chamber of the microfluidic chip by means of syringe pumps. This confocal image was taken 24 h after seeding to allow cells to sediment and adhere to the fibrin matrix. X,Y and Z represent the three axes of projection.  Scale bar: 100 μm.
